# Retained Intraabdominal Gossypiboma, Five Years after Bilateral Orchiopexy

**DOI:** 10.1155/2010/420357

**Published:** 2010-03-04

**Authors:** Mohammad Kazem Moslemi, Mehdi Abedinzadeh

**Affiliations:** ^1^Urology Division, Kamkar Hospital, School of Medicine, Qom Medical Sciences University, 3715694978 Qom, Iran; ^2^Urology Division, Moradi Hospital, Shool of Medicine, Rafsanjan Medical Sciences University, 7713665649 Rafsanjan, Iran

## Abstract

*Introduction.* Gossypiboma or textiloma is used to describe a retained surgical swab in the body after an operation. Intraabdominal surgical sponge is an uncommon surgical error. The incidence of gossypiboma has been reported as high as 1 in 1000 to 15,000 intraabdominal operations. Gossypiboma may cause serious morbidity and may lead to mortality. *Case presentation.* Herein, we report a 24 years-old man who was admitted due to the intraabdominal mass after evaluation of primary infertility. He had a surgical history of bilateral abdominal orchiopexy 5 years previously, performed at another hospital. Hydatid cyst was suspected by abdominal computed tomography. After laparotomy excision, the cyst wall opened incidentally, and draining of a large amount of thick pus with retained surgical gauze within the cyst was found, with final diagnosis of gossypiboma. *Conclusion.* The policy that prevention is far more important than cure is highly appreciated. Accurate sponge and instrument counts, along with radiologic evaluation when a discrepancy is found, can be helpful. Although human errors cannot be completely avoided, continuous medical training and strict adherence to rules of the operation room should reduce the incidence of gossypiboma to a minimum. Surgical sponges should be counted once at the start and twice at the end of all surgical operations.

## 1. Introduction

Gossypiboma is a term used to describe a mass within the body that comprises a cotton matrix surrounded by a foreign body reaction. overlooking a foreign body can sometimes occur despite extreme caution during surgery. A foreign body can trigger a granulomatous reaction and may result in the formation of a sizeable mass. The word “Gossypiboma” is bilingually derived from Latin “gossypium” (cotton) and Kiswahili “boma” (place of concealment) [1].

## 2. Case Presentation

**Figure 1:**
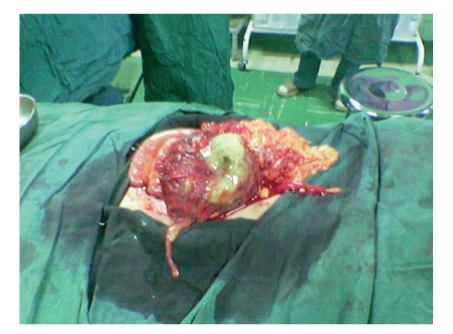
The gauze inside of the cyst, near omentum.

**Figure 2:**
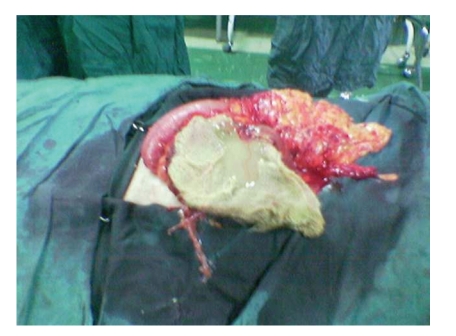
The gauze removed completely from inside of the cyst.

**Figure 3:**
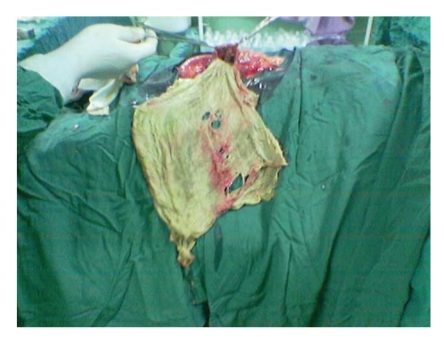
The gauze opened completely.

The patient is a 24-year-old Iranian infertile male that referred to our clinic for work up of infertility. He was a married gentleman since 4 years. In the past medical history no noticeable point is noted. In the past surgical history, he underwent laparotomy for the bilateral orchiopexy of intraabdominal testes 5 years ago in another center. Vital signs were normal and BMI was 22. Upon physical examination, positive findings were atrophic testes and a scar of pfannensteil incision. The findings of laboratory examinations were unremarkable, with a normal cell count and normal biochemistry. Azospermia was noted in the semen analysis. In the hormone prophile, we noted elevated FSH and LH, 2-3 times of normal with normal serum levels of testosterone and prolactin. Due to mild and chronic nonspecific abdominal pain, abdominal ultrasonography was requested, in which a round mass of 5 to 10 cm size with fluid echogenicity is found (in the right lower quadrant). Finally in the abdominopelvic CT scan, a heterogenous soft tissue mass was found in the same reported area of the abdomen near the bladder. He scheduled for laparotomy with hydatid cyst diagnosis. After opening of the layers and peritoneum with low midline incision, the mass was intraperitoneal, that lied between loops of the small intestine. We were very careful about the tearing or rupture of the cyst wall, but because of some adherence it was ruptured and yellow, thick pus released in which a sponge was found (Figures 1, 2, and 3). The operation terminated unevently and the patient was discharged home after 3 days, without any problem.

## 3. Discussion

Retained postoperative foreign body, of which surgical sponges are the most common, is a rare condition. The incidence of gossypiboma is difficult to calculate. It varies between 1 in 100 and 1 in 5,000 procedures [2, 3], because some patients remain asymptomatic and are never discovered. This condition is often underestimated because case numbers are calculated only on the basis of malpractice claims and because the operations that form the denominator for their calculation include large numbers of procedures that are unlikely to result in retained sponges. Another reason of unreporting of occurrences is due to the fear of medicolegal repercussions. It is difficult to recognize a gossypiboma by using radiological screening if the sponge does not have any radiological marker on itself, because the cotton can simulate hematoma, granulomatous process, abscess formation, cystic masses or neoplasm. Gossypiboma can have atypicall calcification and air bubbles as well [4]. Gossipybomas most commonly occur in the abdominal or pelvic cavity, as after gynecologic and upper abdominal surgical procedures [5]. Much of the gossypibomas (75%) are identified only after abdominal or pelvic surgery [4]. Retained surgical sponges can cause serious consequences such as bowel or visceral perforation, obstruction or fistula formation, sepsis or even death [6]. Intraabdominal gossypibomas can migrate into the ileum, stomach, colon or bladder without any apparent opening in the wall of these luminal organs [7]. Retained sponges are more common in obese patients and after emergency surgery [8, 9]. Obese patients have a huge intraperitoneal space to hide sponges, and obesity may increase the technical difficulty of the operation. Gawande et al. reported that retained sponges are 9 times more likely after an emergency operation and 4 times more likely when an unexpected change in the surgical procedure is undertaken [6].

 The clinical presentation of gossypiboma is variable and depends on the location of the sponge and the type of reaction. 

There are 2 types of foreign body reaction in pathology: an exudates reaction leading to abscess formation like our case or chronic internal or external fistula formation, and an aseptic fibrinous reaction resulting in adhesion, encapsulation, and eventual formation of granuloma. The latter usually presents much later than exudates reaction sequelae. They usually remain asymptomatic or present with pseudotumor syndrome [5]. This inflammatory granulomatous reaction is the most likely cause of the extraosseous accumulation of Tc-99m MDP [10].

Common symptoms and signs of gossypiboma are abdominal distention, ileus, tenesmus, pain, palpable mass, vomiting, weight loss, diarrhea, abscess, and fistula formation [11]. Because the symptoms of gossypiboma are usually nonspecific and may appear years after surgery, the diagnosis of gossypiboma usually comes from imaging studies and a high index of suspicion. 

The most impressive imaging finding of gossypiboma is the curved or banded radio-opaque lines on plain radiograph. The ultrasound feature is usually a well-defined mass containing wavy internal echogenic focus with a hypoechoic rim and a strong posterior shadow. However, this is often misinterpreted due to its clinical rarity [12]. On CT, a gossypiboma may manifest as a cystic lesion with internal spongiform appearance with mottled shadows as bubbles, hyperdense capsule, concentric layering, or mottled shadows as bubbles, hyperdense capsule, or mottled mural calcifications [13]. When no radio-opaque marker is seen on X-ray or CT, the characteristic internal structure of the gauze granuloma is best visualized on magnetic resonance imaging. It may appear as a low-signal-intensity lesion on T2-weighted images with wavy, folded fabric inner structure, striped or spotted appearance [14]. 

Possible causatives of sponge retention are emergency surgery, unexpected change in the surgical procedure, disorganization, hurried sponge counts, long operations, unstable patient condition, inexperienced staff, inadequate staff numbers, and patient with high body mass index (BMI) [15]. The present patient was not an obese man because his BMI was 22.

Newer technologies are being developed that will hopefully decrease the incidence of retained foreign body, like radiofrequency identification (RFID). In this system, commonly used surgical gauze sponges, which have been tagged with a radiofrequency identification (RFID) chip scanned with a barcode scanner [16]. The overall objective of this system would be to eliminate errors in the sponge count by removing the human error factor. Furthermore, the sponge count protocol itself has been implicated as a hazard to patient safety [17].

## 4. Conclusion

 Gossypibomas are uncommon, mostly asymptomatic, and hard to diagnose. Particularly chronic cases do not show specific clinical and radiological signs for differential diagnosis. Textiloma should be included in the differential diagnosis of soft-tissue masses detected in patients with a history of a prior operation. Patient-clinician and clinician-radiologist interactions and compliance enhance the possibility of accurate diagnosis.

##  Consent

Written informed consent was obtained from the patient for publication of this case report and accompanying images. A copy of the written consent is available for review by the Editor-in-Chief of this journal.

##  Competing Interests

The authors declare that they have no competing interests.

##  Authors' Contributions

M. K. Moslemi is the corresponding author and responsible for the surgery team. M. Abedinzadeh is the editor of the text and data collector.

## Abbreviations

CT: Computed tomography
BMI: Body mass index
FSH: Follicle stimulating hormone
LH: Luteinizing hormone
MDP: Methylendiphosphate
RFID: Radiofrequency identification.
